# Obesity and Outcome of Assisted Reproduction in Patients With Polycystic Ovary Syndrome

**DOI:** 10.3389/fendo.2018.00149

**Published:** 2018-04-04

**Authors:** Konstantinos Tziomalos, Konstantinos Dinas

**Affiliations:** ^1^First Propedeutic Department of Internal Medicine, Medical School, Aristotle University of Thessaloniki, AHEPA Hospital, Thessaloniki, Greece; ^2^Second Department of Obstetrics and Gynecology, Medical School, Aristotle University of Thessaloniki, Hippokration Hospital, Thessaloniki, Greece

**Keywords:** obesity, polycystic ovary syndrome, weight loss, assisted reproduction, *in vitro* fertilization

## Abstract

Assisted reproduction, including *in vitro* fertilization and intracytoplasmic sperm injection, is increasingly being used for the management of infertility in patients with polycystic ovary syndrome (PCOS). However, there are limited data regarding the association between obesity and the outcome of assisted reproduction in this specific population as well as on the effects of weight loss. The aim of the present review is to summarize the existing evidence on the association between obesity and the outcome of assisted reproduction in patients with PCOS. Accumulating data suggest that obesity is associated with lower pregnancy and live birth rates in patients with PCOS who are undergoing assisted reproduction therapy. However, it remains unclear whether weight loss improves the outcome of this therapy. Notably, recent guidelines state that the health benefits of postponing pregnancy to achieve weight loss must be balanced against the risk of declining fertility with advancing age. Therefore, if weight loss is not achieved within a reasonable time period, assisted reproduction therapy should be offered in adequately selected patients with PCOS, regardless of the presence of obesity.

## Introduction

In recent decades, the prevalence of obesity has increased globally and reached pandemic proportions (1). In high-income countries, approximately one-third of adults are obese and one-third are overweight (1). This rise in the prevalence of obesity has important health-care implications, since obesity is an important risk factor for cardiovascular disease and all-cause mortality (2, 3).

In addition to its cardiometabolic sequelae, obesity is implicated in the pathogenesis of polycystic ovary syndrome (PCOS) (4, 5). Approximately 40–70% of patients with this syndrome are either overweight or obese (4–6). The prevalence of PCOS is also almost four times higher in overweight and obese patients than in lean subjects (7). However, other studies reported that obesity only minimally increases the risk of PCOS (4, 8). Moreover, it has been reported that PCOS might also increase the risk of obesity, potentially by a reduction in basal metabolic rate and an impairment in appetite regulation (9–11).

Polycystic ovary syndrome is the commonest endocrine disorder in women of reproductive age (8, 12, 13) and the leading cause of anovulatory infertility (14). Several studies showed that obese patients with PCOS have more impaired ovulation and lower pregnancy rates than normal-weight patients with this syndrome (15–17).

Assisted reproduction, including *in vitro* fertilization (IVF) and intracytoplasmic sperm injection (ICSI), is increasingly being used for the management of infertility in patients with PCOS (14). However, there are limited data regarding the association between obesity and the outcome of assisted reproduction in this specific population as well as on the effects of weight loss. The aim of the present review is to summarize the existing evidence on the association between obesity and the outcome of assisted reproduction in patients with PCOS.

## Effects of Obesity on the Outcome of Assisted Reproduction in Patients with PCOS

A number of studies evaluated the association between obesity and the outcome of assisted reproduction in patients with PCOS (Table 1). However, most studies were small and retrospective. Moreover, most studies included patients with various causes of fertility and did not perform separate analyses of patients with PCOS.

**Table 1:** Studies evaluating the association between obesity and the outcome of assisted reproduction in patients with polycystic ovary syndrome.

Reference	*n*	Outcome
(18)	16	Retrieved oocytes (lean and obese patients): 22.2 ± 9.2 vs. 14.3 ± 4.9 (*p* = 0.04)
		Implantation rates: 0.55 ± 0.39 vs. 0.20 ± 0.37 (*p* = NS)
		Live birth rates: 83.3 vs. 45.5% (*p* = NS)

(19)	72	Clinical pregnancy rates (patients with BMI ≥ 40 kg/m^2^ and <40 kg/m^2^): 32 vs. 72% (RR 0.44, 95% CI 0.22–0.87)
		Live birth rates: 32 vs. 60% (RR 0.52, 95% CI 0.26–1.05)

(20)	55	Implantation rates (patients with BMI < 18.5, 18.5–24.9, 25.0–29.9 and ≥30 kg/m^2^): 75.0, 50.7, 57.1 and 27.3% (*p* = 0.016)
		Ongoing pregnancy rates: 100.0, 68.8, 66.7, and 41.7% (*p* = 0.039)

(21)	79	Clinical pregnancy per cycle start (patients with BMI 18.7–24.9 and ≥30 kg/m^2^): 56.9 vs. 35.5% (OR 0.31, 95% CI 0.11–0.86)
		Live birth per cycle start: 49.0 vs. 32.3% (OR 0.29, 95% CI 0.10–0.84)

(22)	100	Fertilization rates (patients with BMI ≤ 25 and >25 kg/m^2^): 62 ± 18 vs. 44 ± 22 (*p* = 0.02)
		Clinical pregnancy rates: 44.4 vs. 11.8% (*p* = 0.02)

*BMI, body mass index; RR, relative risk; CI, confidence interval; OR: odds ratio*.

In a small retrospective study (*n* = 16), lean patients with PCOS had less retrieved oocytes than obese patients with PCOS, even though they required fewer gonadotropin ampoules (18). In contrast, implantation, clinical pregnancy and live birth rates did not differ between the two groups, possibly due to the small number of patients (18). Indeed, in a larger retrospective study (*n* = 72), patients with PCOS and body mass index (BMI) ≥ 40 kg/m^2^ had significantly lower clinical pregnancy rates after IVF than patients with PCOS and lower BMI (32 vs. 72%, respectively) and a trend for lower live birth rates (32 vs. 60%, respectively) despite the absence of difference in number of embryos transferred and implantation rates between the two groups (19). Moreover, the former required higher doses of gonadotropin and had fewer oocytes retrieved than the latter (19). In another study that included 55 patients with PCOS, implantation and pregnancy rates declined with increasing BMI (20). More specifically, implantation rates in patients with BMI < 18.5, 18.5–24.9, 25.0–29.9, and ≥30 kg/m^2^ were 75.0, 50.7, 57.1, and 27.3%, respectively (*p* = 0.016) whereas ongoing pregnancy rates were 100.0, 68.8, 66.7, and 41.7%, respectively (*p* = 0.039) (20). Notably, the total gonadotropin dose and the number of oocytes retrieved did not differ between the different BMI categories (20).

In a retrospective study in 79 patients with PCOS undergoing IVF, obese patients with PCOS had 69% lower odds of clinical pregnancy per cycle start than patients with PCOS and normal BMI (*p* = 0.02) and 77% lower odds of clinical pregnancy per embryo transfer (*p* = 0.008) (21). In addition, the odds of live birth were 71% lower in obese patients per cycle start (*p* = 0.02) and 77% lower per embryo transfer (*p* = 0.01) (21). On the other hand, a trend for lower incidence of ovarian hyperstimulation syndrome (OHSS) was observed (19.6, 10.5, and 3.2% in normal weight, overweight, and obese patients, respectively) (21).

In the largest study performed specifically in patients with PCOS (*n* = 100), patients with PCOS and BMI > 25 kg/m^2^ undergoing IVF had lower fertilization rates than patients with PCOS and BMI ≤ 25 kg/m^2^ undergoing IVF (44 ± 22 vs. 62 ± 18, respectively) and also had lower clinical pregnancy rates (11.8 vs. 44.4%, respectively) (22). Interestingly, these differences were present in both patients undergoing ovarian stimulation with gonadotrophin-releasing hormone (GnRH) agonist and in those undergoing stimulation with GnRH antagonist (22). Of note, GnRH agonists prevent premature luteinizing hormone (LH) surge, thereby increasing the number of retrieved oocytes and pregnancy rates and decreasing the number of cycle cancelations (23). However, they might increase the risk for OHSS (23). On the other hand, GnRH antagonists can competitively block GnRH receptors and cause rapid suppression of gonadotropin release, resulting in fewer complications but appear to be less effective than GnRH agonists (23). Notably, follicle-stimulating hormone (FSH) preparations might offer a more physiologic approach in patients with PCOS, since the LH/FSH ratio is frequently elevated in this population (24).

There are very limited data on the association between obesity and the outcomes of ICSI in patients with PCOS. In a small study in 56 patients with PCOS undergoing IVF or ICSI, obesity was independently related to a lower oocyte count and increased FSH requirement (25). However, pregnancy and live birth rates were not reported and outcomes were not reported separately in patients receiving IVF or ICSI (25).

## Effects of Weight Loss on the Outcome of Assisted Reproduction in Patients with PCOS

Several small (*n* = 18–67) and uncontrolled studies showed that lifestyle-induced weight loss restores ovulation in patients with PCOS (26–29). Weight loss also increased spontaneous pregnancy rates in these studies (27, 28). Case series also suggested that patients with PCOS undergoing bariatric surgery were able to conceive postoperatively (30, 31).

There are very few randomized controlled study (RCTs) that evaluated the effects of weight loss on the outcome of assisted reproduction in patients with PCOS. An early RCT in 38 patients with various causes of infertility showed no benefit of diet and exercise-induced weight loss on pregnancy and live birth rates (32). However, weight loss was modest (3.8 kg) and the change in waist circumference was similar in patients who implemented lifestyle changes and in controls (32). In contrast, a more recent and larger RCT (*n* = 49) reported higher pregnancy and live birth rates in patients with various causes of infertility who lost approximately 6.6 kg with diet, exercise, and behavioral modification (33). Notably, fewer ART cycles were required to achieve these higher rates in patients who lost weight (33). Nevertheless, neither of these studies reported the effects of weight loss in the subgroup of patients with PCOS (32, 33).

In a secondary analysis of the pregnancy in PPCOS II trial (*n* = 187) and the treatment of hyperandrogenism versus insulin resistance in infertile PCOS (OWL PCOS) trial (*n* = 142), lifestyle modification (caloric restriction, antiobesity medication, behavioral modification, and exercise) followed by treatment with clomiphene resulted in higher rates of ovulation and live birth compared with immediate treatment with clomiphene (risk ratio 1.4 and 2.5, respectively) (34). Of note, clomiphene is the treatment of first choice for induction of ovulation in women with PCOS (35, 36). Clomiphene induces ovulation through its anti-estrogen action, which results in a change in GnRH pulse frequency, release of FSH from the anterior pituitary and consequent follicular development (24). Treatment with clomiphene results in ovulation in 75–80% of patients and increases the likelihood of live birth approximately six times more than placebo and three times more than metformin (35–38).

The promising findings of the latter studies were not confirmed in the largest RCT that evaluated the role of weight loss in infertile patients receiving assisted reproduction treatment. In this study, 577 infertile women (201 with PCOS) were randomized to receive a 6-month lifestyle-intervention program followed by 18 months of infertility treatment or prompt infertility treatment for 24 months (39). The primary outcome (vaginal birth of a healthy singleton at term within 24 months after randomization) occurred in a smaller percentage of the women who followed a lifestyle program than in those who received prompt infertility treatment (27.1 vs. 35.2%, respectively; rate ratio 0.77, 95% confidence interval 0.60–0.99, *p* = 0.04) (39). However, weight loss was rather small (4.4 kg) and only 37.7% of patients randomized to lifestyle changes lost >5% of their body weight (39). Moreover, a considerable proportion of patients in the lifestyle arm discontinued treatment (21.8%) (39). Importantly, rates of pregnancy resulting from natural conception were higher in women assigned lifestyle changes (26.1 vs. 16.2% in patients assigned prompt infertility treatment) (39). Accordingly, the use of ovulation induction or other infertility treatment was less frequent and the number of infertility treatment cycles was lower in the lifestyle arm (39). Subgroup analyses among the 201 PCOS patients that were included in this study were not reported (39). However, among women with anovulatory infertility (*n* = 269), the rates of the primary outcome, live birth, and ongoing pregnancy did not differ between the intervention and control group (39).

## Conclusion

Accumulating data suggest that obesity is associated with lower pregnancy and live birth rates in patients with PCOS who are undergoing assisted reproduction therapy. However, it remains unclear whether weight loss improves the outcome of this therapy. Notably, the American Society for Reproductive Medicine recently concluded that the health benefits of postponing pregnancy to achieve weight loss must be balanced against the risk of declining fertility with advancing age (40). Therefore, if weight loss is not achieved within a reasonable time period, assisted reproduction therapy should be offered in adequately selected patients with PCOS, regardless of the presence of obesity.

## Author Contributions

KT drafted the mini review. KD critically revised the draft.

## Conflict of Interest Statement

The authors declare that the research was conducted in the absence of any commercial or financial relationships that could be construed as a potential conflict of interest.
